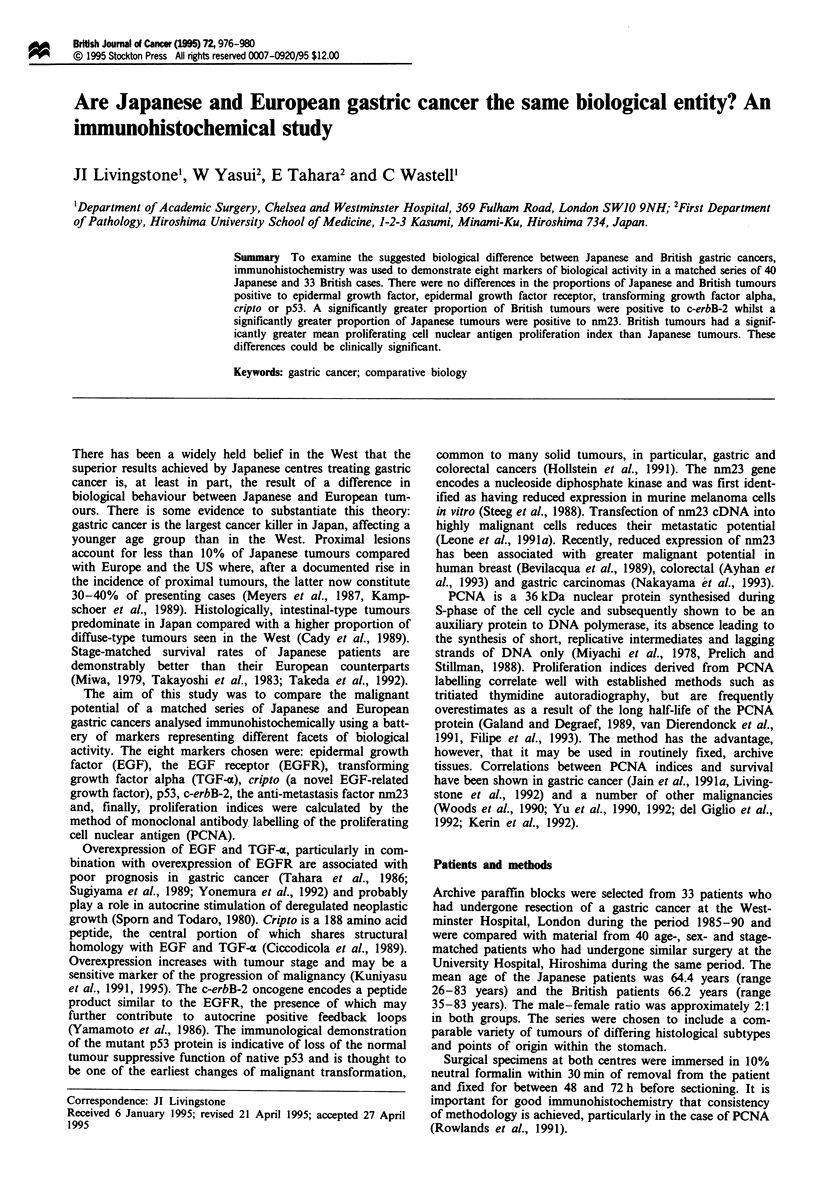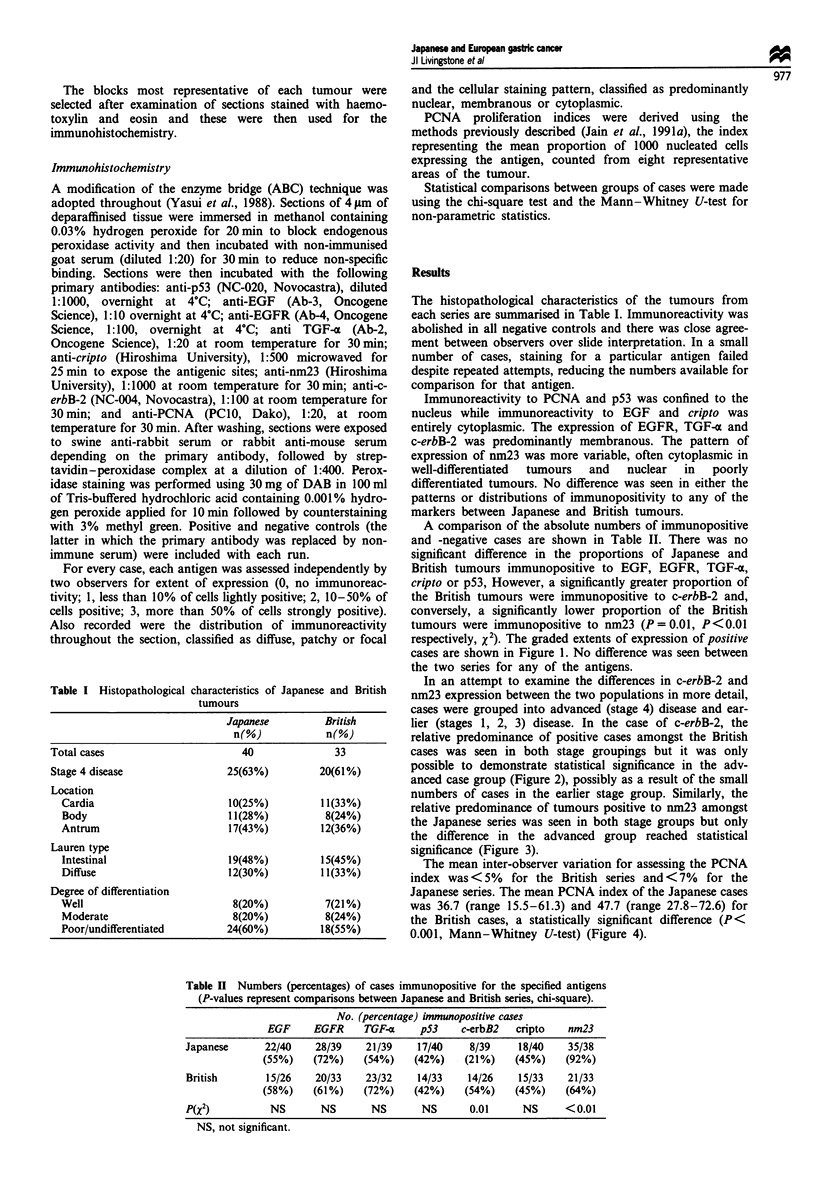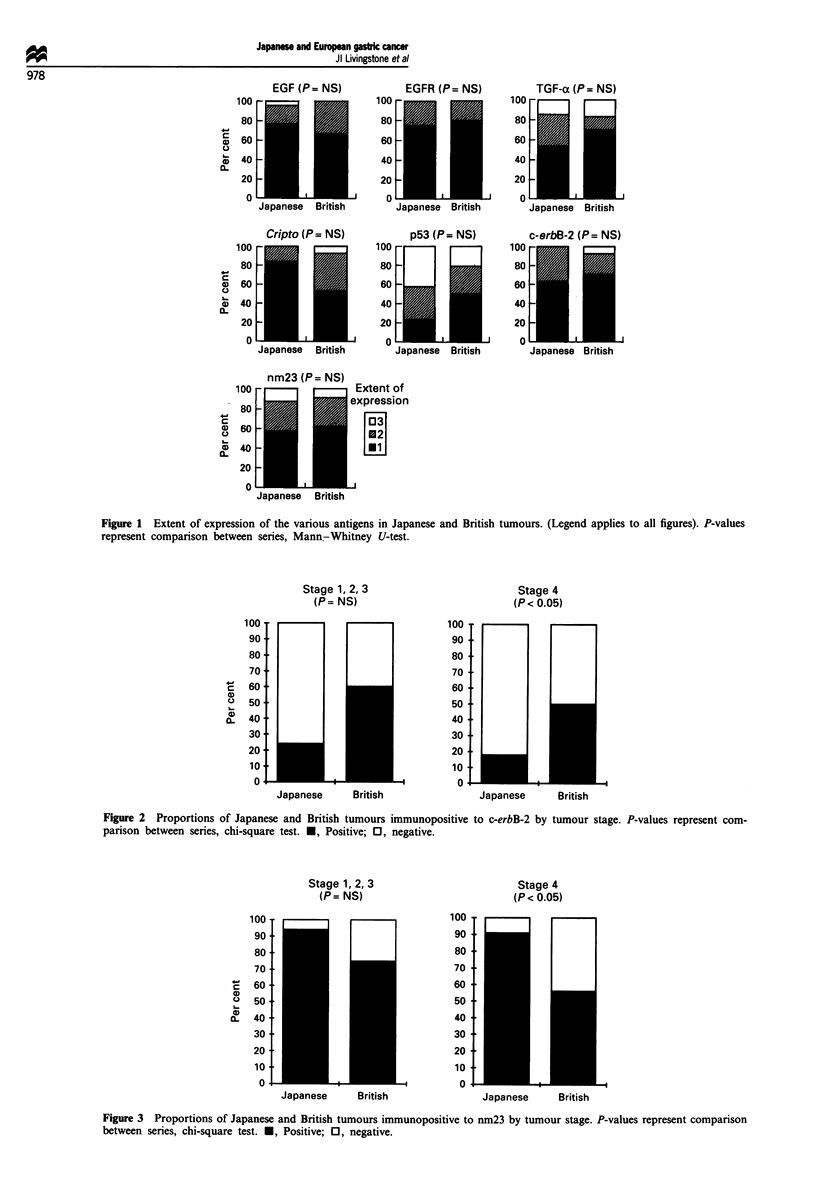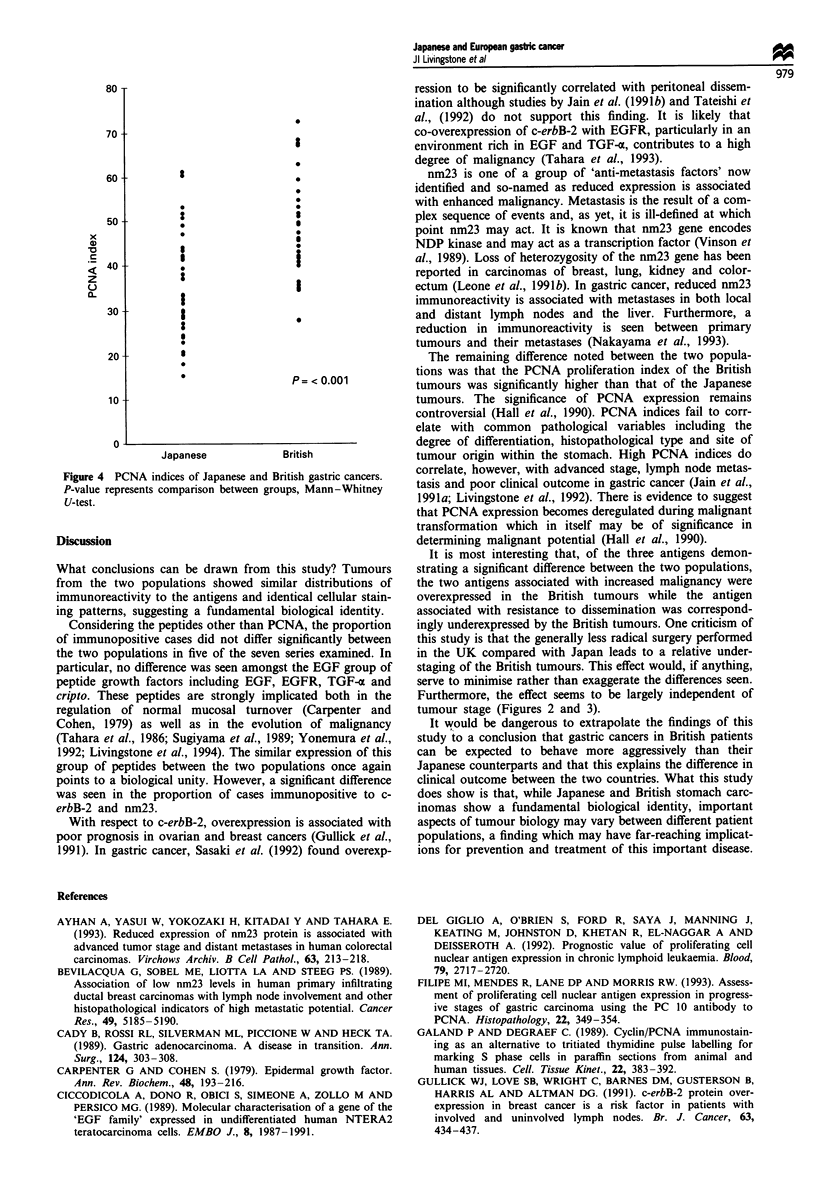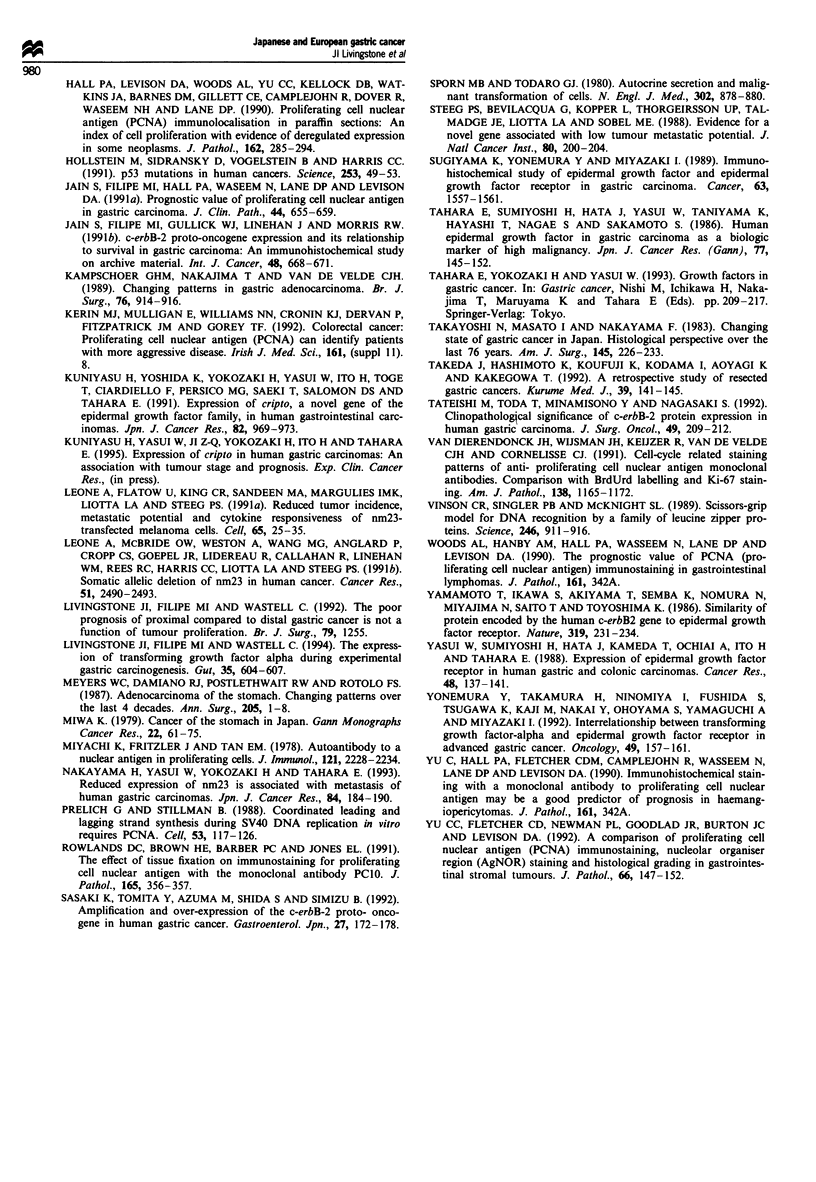# Are Japanese and European gastric cancer the same biological entity? An immunohistochemical study.

**DOI:** 10.1038/bjc.1995.445

**Published:** 1995-10

**Authors:** J. I. Livingstone, W. Yasui, E. Tahara, C. Wastell

**Affiliations:** Department of Academic Surgery, Chelsea and Westminster Hospital, London.

## Abstract

To examine the suggested biological difference between Japanese and British gastric cancers, immunohistochemistry was used to demonstrate eight markers of biological activity in a matched series of 40 Japanese and 33 British cases. There were no differences in the proportions of Japanese and British tumours positive to epidermal growth factor, epidermal growth factor receptor, transforming growth factor alpha, cripto or p53. A significantly greater proportion of British tumours were positive to c-erbB-2 whilst a significantly greater proportion of Japanese tumours were positive to nm23. British tumours had a significantly greater mean proliferating cell nuclear antigen proliferation index than Japanese tumours. These differences could be clinically significant.


British Journal of Cancer (1995) 72, 976-980
© 1995 Stockton Press All rights reserved 0007-0920/95 $12.00

### Are Japanese and European gastric cancer the same biological entity? An immunohistochemical study

JI Livingstone[1], W Yasui[2], E Tahara[2] and C Wastell[1]

[1]*Department of Academic Surgery, Chelsea and Westminster Hospital, 369 Fulham Road, London SW10 9NH;* [2]*First Department of Pathology, Hiroshima University School of Medicine, 1-2-3 Kasumi, Minami-Ku, Hiroshima 734, Japan.*

**Summary** To examine the suggested biological difference between Japanese and British gastric cancers, immunohistochemistry was used to demonstrate eight markers of biological activity in a matched series of 40 Japanese and 33 British cases. There were no differences in the proportions of Japanese and British tumours positive to epidermal growth factor, epidermal growth factor receptor, transforming growth factor alpha, *cripto* or p53. A significantly greater proportion of British tumours were positive to c-*erb*B-2 whilst a significantly greater proportion of Japanese tumours were positive to nm23. British tumours had a significantly greater mean proliferating cell nuclear antigen proliferation index than Japanese tumours. These differences could be clinically significant.

**Keywords:** gastric cancer; comparative biology

There has been a widely held belief in the West that the superior results achieved by Japanese centres treating gastric cancer is, at least in part, the result of a difference in biological behaviour between Japanese and European tumours. There is some evidence to substantiate this theory: gastric cancer is the largest cancer killer in Japan, affecting a younger age group than in the West. Proximal lesions account for less than 10% of Japanese tumours compared with Europe and the US where, after a documented rise in the incidence of proximal tumours, the latter now constitute 30–40% of presenting cases (Meyers *et al.*, 1987, Kampschoer *et al.*, 1989). Histologically, intestinal-type tumours predominate in Japan compared with a higher proportion of diffuse-type tumours seen in the West (Cady *et al.*, 1989). Stage-matched survival rates of Japanese patients are demonstrably better than their European counterparts (Miwa, 1979, Takayoshi *et al.*, 1983; Takeda *et al.*, 1992).

The aim of this study was to compare the malignant potential of a matched series of Japanese and European gastric cancers analysed immunohistochemically using a battery of markers representing different facets of biological activity. The eight markers chosen were: epidermal growth factor (EGF), the EGF receptor (EGFR), transforming growth factor alpha (TGF-α), *cripto* (a novel EGF-related growth factor), p53, c-*erb*B-2, the anti-metastasis factor nm23 and, finally, proliferation indices were calculated by the method of monoclonal antibody labelling of the proliferating cell nuclear antigen (PCNA).

Overexpression of EGF and TGF-α, particularly in combination with overexpression of EGFR are associated with poor prognosis in gastric cancer (Tahara *et al.*, 1986; Sugiyama *et al.*, 1989; Yonemura *et al.*, 1992) and probably play a role in autocrine stimulation of deregulated neoplastic growth (Sporn and Todaro, 1980). *Cripto* is a 188 amino acid peptide, the central portion of which shares structural homology with EGF and TGF-α (Ciccodicola *et al.*, 1989). Overexpression increases with tumour stage and may be a sensitive marker of the progression of malignancy (Kuniyasu *et al.*, 1991, 1995). The c-*erb*B-2 oncogene encodes a peptide product similar to the EGFR, the presence of which may further contribute to autocrine positive feedback loops (Yamamoto *et al.*, 1986). The immunological demonstration of the mutant p53 protein is indicative of loss of the normal tumour suppressive function of native p53 and is thought to be one of the earliest changes of malignant transformation, common to many solid tumours, in particular, gastric and colorectal cancers (Hollstein *et al.*, 1991). The nm23 gene encodes a nucleoside diphosphate kinase and was first identified as having reduced expression in murine melanoma cells *in vitro* (Steeg *et al.*, 1988). Transfection of nm23 cDNA into highly malignant cells reduces their metastatic potential (Leone *et al.*, 1991*a*). Recently, reduced expression of nm23 has been associated with greater malignant potential in human breast (Bevilacqua *et al.*, 1989), colorectal (Ayhan *et al.*, 1993) and gastric carcinomas (Nakayama *et al.*, 1993).

PCNA is a 36 kDa nuclear protein synthesised during S-phase of the cell cycle and subsequently shown to be an auxiliary protein to DNA polymerase, its absence leading to the synthesis of short, replicative intermediates and lagging strands of DNA only (Miyachi *et al.*, 1978, Prelich and Stillman, 1988). Proliferation indices derived from PCNA labelling correlate well with established methods such as tritiated thymidine autoradiography, but are frequently overestimates as a result of the long half-life of the PCNA protein (Galand and Degraef, 1989, van Dierendonck *et al.*, 1991, Filipe *et al.*, 1993). The method has the advantage, however, that it may be used in routinely fixed, archive tissues. Correlations between PCNA indices and survival have been shown in gastric cancer (Jain *et al.*, 1991*a*, Livingstone *et al.*, 1992) and a number of other malignancies (Woods *et al.*, 1990; Yu *et al.*, 1990, 1992; del Giglio *et al.*, 1992; Kerin *et al.*, 1992).

### Patients and methods

Archive paraffin blocks were selected from 33 patients who had undergone resection of a gastric cancer at the Westminster Hospital, London during the period 1985–90 and were compared with material from 40 age-, sex- and stage-matched patients who had undergone similar surgery at the University Hospital, Hiroshima during the same period. The mean age of the Japanese patients was 64.4 years (range 26–83 years) and the British patients 66.2 years (range 35–83 years). The male–female ratio was approximately 2:1 in both groups. The series were chosen to include a comparable variety of tumours of differing histological subtypes and points of origin within the stomach.

Surgical specimens at both centres were immersed in 10% neutral formalin within 30 min of removal from the patient and fixed for between 48 and 72 h before sectioning. It is important for good immunohistochemistry that consistency of methodology is achieved, particularly in the case of PCNA (Rowlands *et al.*, 1991).

Correspondence: JI Livingstone
Received 6 January 1995; revised 21 April 1995; accepted 27 April 1995

The blocks most representative of each tumour were selected after examination of sections stained with haemotoxylin and eosin and these were then used for the immunohistochemistry.

*Immunohistochemistry*

A modification of the enzyme bridge (ABC) technique was adopted throughout (Yasui *et al.*, 1988). Sections of 4 μm of deparaffinised tissue were immersed in methanol containing 0.03% hydrogen peroxide for 20 min to block endogenous peroxidase activity and then incubated with non-immunised goat serum (diluted 1:20) for 30 min to reduce non-specific binding. Sections were then incubated with the following primary antibodies: anti-p53 (NC-020, Novocastra), diluted 1:1000, overnight at 4°C; anti-EGF (Ab-3, Oncogene Science), 1:10 overnight at 4°C; anti-EGFR (Ab-4, Oncogene Science, 1:100, overnight at 4°C; anti TGF-α (Ab-2, Oncogene Science), 1:20 at room temperature for 30 min; anti-*cripto* (Hiroshima University), 1:500 microwaved for 25 min to expose the antigenic sites; anti-nm23 (Hiroshima University), 1:1000 at room temperature for 30 min; anti-c-*erb*B-2 (NC-004, Novocastra), 1:100 at room temperature for 30 min; and anti-PCNA (PC10, Dako), 1:20, at room temperature for 30 min. After washing, sections were exposed to swine anti-rabbit serum or rabbit anti-mouse serum depending on the primary antibody, followed by streptavidin–peroxidase complex at a dilution of 1:400. Peroxidase staining was performed using 30 mg of DAB in 100 ml of Tris-buffered hydrochloric acid containing 0.001% hydrogen peroxide applied for 10 min followed by counterstaining with 3% methyl green. Positive and negative controls (the latter in which the primary antibody was replaced by non-immune serum) were included with each run.

For every case, each antigen was assessed independently by two observers for extent of expression (0, no immunoreactivity; 1, less than 10% of cells lightly positive; 2, 10–50% of cells positive; 3, more than 50% of cells strongly positive). Also recorded were the distribution of immunoreactivity throughout the section, classified as diffuse, patchy or focal and the cellular staining pattern, classified as predominantly nuclear, membranous or cytoplasmic.

PCNA proliferation indices were derived using the methods previously described (Jain *et al.*, 1991*a*), the index representing the mean proportion of 1000 nucleated cells expressing the antigen, counted from eight representative areas of the tumour.

Statistical comparisons between groups of cases were made using the chi-square test and the Mann–Whitney *U*-test for non-parametric statistics.

**Table I** Histopathological characteristics of Japanese and British tumours

| | *Japanese* n(%) | *British* n(%) |
|---|---|---|
| Total cases | 40 | 33 |
| Stage 4 disease | 25(63%) | 20(61%) |
| Location | | |
| Cardia | 10(25%) | 11(33%) |
| Body | 11(28%) | 8(24%) |
| Antrum | 17(43%) | 12(36%) |
| Lauren type | | |
| Intestinal | 19(48%) | 15(45%) |
| Diffuse | 12(30%) | 11(33%) |
| Degree of differentiation | | |
| Well | 8(20%) | 7(21%) |
| Moderate | 8(20%) | 8(24%) |
| Poor/undifferentiated | 24(60%) | 18(55%) |

### Results

The histopathological characteristics of the tumours from each series are summarised in Table I. Immunoreactivity was abolished in all negative controls and there was close agreement between observers over slide interpretation. In a small number of cases, staining for a particular antigen failed despite repeated attempts, reducing the numbers available for comparison for that antigen.

Immunoreactivity to PCNA and p53 was confined to the nucleus while immunoreactivity to EGF and *cripto* was entirely cytoplasmic. The expression of EGFR, TGF-α and c-*erb*B-2 was predominantly membranous. The pattern of expression of nm23 was more variable, often cytoplasmic in well-differentiated tumours and nuclear in poorly differentiated tumours. No difference was seen in either the patterns or distributions of immunopositivity to any of the markers between Japanese and British tumours.

A comparison of the absolute numbers of immunopositive and -negative cases are shown in Table II. There was no significant difference in the proportions of Japanese and British tumours immunopositive to EGF, EGFR, TGF-α, *cripto* or p53, However, a significantly greater proportion of the British tumours were immunopositive to c-*erb*B-2 and, conversely, a significantly lower proportion of the British tumours were immunopositive to nm23 ($P = 0.01$, $P < 0.01$ respectively, $\chi^2$). The graded extents of expression of *positive* cases are shown in Figure 1. No difference was seen between the two series for any of the antigens.

In an attempt to examine the differences in c-*erb*B-2 and nm23 expression between the two populations in more detail, cases were grouped into advanced (stage 4) disease and earlier (stages 1, 2, 3) disease. In the case of c-*erb*B-2, the relative predominance of positive cases amongst the British cases was seen in both stage groupings but it was only possible to demonstrate statistical significance in the advanced case group (Figure 2), possibly as a result of the small numbers of cases in the earlier stage group. Similarly, the relative predominance of tumours positive to nm23 amongst the Japanese series was seen in both stage groups but only the difference in the advanced group reached statistical significance (Figure 3).

The mean inter-observer variation for assessing the PCNA index was $<5\%$ for the British series and $<7\%$ for the Japanese series. The mean PCNA index of the Japanese cases was 36.7 (range 15.5–61.3) and 47.7 (range 27.8–72.6) for the British cases, a statistically significant difference ($P < 0.001$, Mann–Whitney *U*-test) (Figure 4).

**Table II** Numbers (percentages) of cases immunopositive for the specified antigens (*P*-values represent comparisons between Japanese and British series, chi-square).

| | *No. (percentage) immunopositive cases* | | | | | | |
|---|---|---|---|---|---|---|---|
| | *EGF* | *EGFR* | *TGF-α* | *p53* | c-*erbB2* | cripto | *nm23* |
| Japanese | 22/40 (55%) | 28/39 (72%) | 21/39 (54%) | 17/40 (42%) | 8/39 (21%) | 18/40 (45%) | 35/38 (92%) |
| British | 15/26 (58%) | 20/33 (61%) | 23/32 (72%) | 14/33 (42%) | 14/26 (54%) | 15/33 (45%) | 21/33 (64%) |
| $P(\chi^2)$ | NS | NS | NS | NS | 0.01 | NS | <0.01 |

NS, not significant.

**Figure 1** Extent of expression of the various antigens in Japanese and British tumours. (Legend applies to all figures). P-values represent comparison between series, Mann–Whitney U-test.

**Figure 2** Proportions of Japanese and British tumours immunopositive to c-*erb*B-2 by tumour stage. P-values represent comparison between series, chi-square test. ■, Positive; □, negative.

**Figure 3** Proportions of Japanese and British tumours immunopositive to nm23 by tumour stage. P-values represent comparison between series, chi-square test. ■, Positive; □, negative.

**Figure 4** PCNA indices of Japanese and British gastric cancers. P-value represents comparison between groups, Mann–Whitney U-test.

### Discussion

What conclusions can be drawn from this study? Tumours from the two populations showed similar distributions of immunoreactivity to the antigens and identical cellular staining patterns, suggesting a fundamental biological identity.

Considering the peptides other than PCNA, the proportion of immunopositive cases did not differ significantly between the two populations in five of the seven series examined. In particular, no difference was seen amongst the EGF group of peptide growth factors including EGF, EGFR, TGF-α and *cripto*. These peptides are strongly implicated both in the regulation of normal mucosal turnover (Carpenter and Cohen, 1979) as well as in the evolution of malignancy (Tahara *et al.*, 1986; Sugiyama *et al.*, 1989; Yonemura *et al.*, 1992; Livingstone *et al.*, 1994). The similar expression of this group of peptides between the two populations once again points to a biological unity. However, a significant difference was seen in the proportion of cases immunopositive to c-*erb*B-2 and nm23.

With respect to c-*erb*B-2, overexpression is associated with poor prognosis in ovarian and breast cancers (Gullick *et al.*, 1991). In gastric cancer, Sasaki *et al.* (1992) found overexpression to be significantly correlated with peritoneal dissemination although studies by Jain *et al.* (1991*b*) and Tateishi *et al.*, (1992) do not support this finding. It is likely that co-overexpression of c-*erb*B-2 with EGFR, particularly in an environment rich in EGF and TGF-α, contributes to a high degree of malignancy (Tahara *et al.*, 1993).

nm23 is one of a group of 'anti-metastasis factors' now identified and so-named as reduced expression is associated with enhanced malignancy. Metastasis is the result of a complex sequence of events and, as yet, it is ill-defined at which point nm23 may act. It is known that nm23 gene encodes NDP kinase and may act as a transcription factor (Vinson *et al.*, 1989). Loss of heterozygosity of the nm23 gene has been reported in carcinomas of breast, lung, kidney and colorectum (Leone *et al.*, 1991*b*). In gastric cancer, reduced nm23 immunoreactivity is associated with metastases in both local and distant lymph nodes and the liver. Furthermore, a reduction in immunoreactivity is seen between primary tumours and their metastases (Nakayama *et al.*, 1993).

The remaining difference noted between the two populations was that the PCNA proliferation index of the British tumours was significantly higher than that of the Japanese tumours. The significance of PCNA expression remains controversial (Hall *et al.*, 1990). PCNA indices fail to correlate with common pathological variables including the degree of differentiation, histopathological type and site of tumour origin within the stomach. High PCNA indices do correlate, however, with advanced stage, lymph node metastasis and poor clinical outcome in gastric cancer (Jain *et al.*, 1991*a*; Livingstone *et al.*, 1992). There is evidence to suggest that PCNA expression becomes deregulated during malignant transformation which in itself may be of significance in determining malignant potential (Hall *et al.*, 1990).

It is most interesting that, of the three antigens demonstrating a significant difference between the two populations, the two antigens associated with increased malignancy were overexpressed in the British tumours while the antigen associated with resistance to dissemination was correspondingly underexpressed by the British tumours. One criticism of this study is that the generally less radical surgery performed in the UK compared with Japan leads to a relative understaging of the British tumours. This effect would, if anything, serve to minimise rather than exaggerate the differences seen. Furthermore, the effect seems to be largely independent of tumour stage (Figures 2 and 3).

It would be dangerous to extrapolate the findings of this study to a conclusion that gastric cancers in British patients can be expected to behave more aggressively than their Japanese counterparts and that this explains the difference in clinical outcome between the two countries. What this study does show is that, while Japanese and British stomach carcinomas show a fundamental biological identity, important aspects of tumour biology may vary between different patient populations, a finding which may have far-reaching implications for prevention and treatment of this important disease.

### References

AYHAN A, YASUI W, YOKOZAKI H, KITADAI Y AND TAHARA E. (1993). Reduced expression of nm23 protein is associated with advanced tumor stage and distant metastases in human colorectal carcinomas. *Virchows Archiv. B Cell Pathol.*, **63**, 213–218.

BEVILACQUA G, SOBEL ME, LIOTTA LA AND STEEG PS. (1989). Association of low nm23 levels in human primary infiltrating ductal breast carcinomas with lymph node involvement and other histopathological indicators of high metastatic potential. *Cancer Res.*, **49**, 5185–5190.

CADY B, ROSSI RL, SILVERMAN ML, PICCIONE W AND HECK TA. (1989). Gastric adenocarcinoma. A disease in transition. *Ann. Surg.*, **124**, 303–308.

CARPENTER G AND COHEN S. (1979). Epidermal growth factor. *Ann. Rev. Biochem.*, **48**, 193–216.

CICCODICOLA A, DONO R, OBICI S, SIMEONE A, ZOLLO M AND PERSICO MG. (1989). Molecular characterisation of a gene of the 'EGF family' expressed in undifferentiated human NTERA2 teratocarcinoma cells. *EMBO J.*, **8**, 1987–1991.

DEL GIGLIO A, O'BRIEN S, FORD R, SAYA J, MANNING J, KEATING M, JOHNSTON D, KHETAN R, EL-NAGGAR A AND DEISSEROTH A. (1992). Prognostic value of proliferating cell nuclear antigen expression in chronic lymphoid leukaemia. *Blood*, **79**, 2717–2720.

FILIPE MI, MENDES R, LANE DP AND MORRIS RW. (1993). Assessment of proliferating cell nuclear antigen expression in progressive stages of gastric carcinoma using the PC 10 antibody to PCNA. *Histopathology*, **22**, 349–354.

GALAND P AND DEGRAEF C. (1989). Cyclin/PCNA immunostaining as an alternative to tritiated thymidine pulse labelling for marking S phase cells in paraffin sections from animal and human tissues. *Cell. Tissue Kinet.*, **22**, 383–392.

GULLICK WJ, LOVE SB, WRIGHT C, BARNES DM, GUSTERSON B, HARRIS AL AND ALTMAN DG. (1991). c-*erb*B-2 protein overexpression in breast cancer is a risk factor in patients with involved and uninvolved lymph nodes. *Br. J. Cancer*, **63**, 434–437.

HALL PA, LEVISON DA, WOODS AL, YU CC, KELLOCK DB, WATKINS JA, BARNES DM, GILLETT CE, CAMPLEJOHN R, DOVER R, WASEEM NH AND LANE DP. (1990). Proliferating cell nuclear antigen (PCNA) immunolocalisation in paraffin sections: An index of cell proliferation with evidence of deregulated expression in some neoplasms. *J. Pathol.*, **162**, 285–294.

HOLLSTEIN M, SIDRANSKY D, VOGELSTEIN B AND HARRIS CC. (1991). p53 mutations in human cancers. *Science*, **253**, 49–53.

JAIN S, FILIPE MI, HALL PA, WASEEM N, LANE DP AND LEVISON DA. (1991*a*). Prognostic value of proliferating cell nuclear antigen in gastric carcinoma. *J. Clin. Path.*, **44**, 655–659.

JAIN S, FILIPE MI, GULLICK WJ, LINEHAN J AND MORRIS RW. (1991*b*). c-*erb*B-2 proto-oncogene expression and its relationship to survival in gastric carcinoma: An immunohistochemical study on archive material. *Int. J. Cancer*, **48**, 668–671.

KAMPSCHOER GHM, NAKAJIMA T AND VAN DE VELDE CJH. (1989). Changing patterns in gastric adenocarcinoma. *Br. J. Surg.*, **76**, 914–916.

KERIN MJ, MULLIGAN E, WILLIAMS NN, CRONIN KJ, DERVAN P, FITZPATRICK JM AND GOREY TF. (1992). Colorectal cancer: Proliferating cell nuclear antigen (PCNA) can identify patients with more aggressive disease. *Irish J. Med. Sci.*, **161**, (suppl 11). 8.

KUNIYASU H, YOSHIDA K, YOKOZAKI H, YASUI W, ITO H, TOGE T, CIARDIELLO F, PERSICO MG, SAEKI T, SALOMON DS AND TAHARA E. (1991). Expression of *cripto*, a novel gene of the epidermal growth factor family, in human gastrointestinal carcinomas. *Jpn. J. Cancer Res.*, **82**, 969–973.

KUNIYASU H, YASUI W, JI Z-Q, YOKOZAKI H, ITO H AND TAHARA E. (1995). Expression of *cripto* in human gastric carcinomas: An association with tumour stage and prognosis. *Exp. Clin. Cancer Res.*, (in press).

LEONE A, FLATOW U, KING CR, SANDEEN MA, MARGULIES IMK, LIOTTA LA AND STEEG PS. (1991*a*). Reduced tumor incidence, metastatic potential and cytokine responsiveness of nm23-transfected melanoma cells. *Cell*, **65**, 25–35.

LEONE A, McBRIDE OW, WESTON A, WANG MG, ANGLARD P, CROPP CS, GOEPEL JR, LIDEREAU R, CALLAHAN R, LINEHAN WM, REES RC, HARRIS CC, LIOTTA LA AND STEEG PS. (1991*b*). Somatic allelic deletion of nm23 in human cancer. *Cancer Res.*, **51**, 2490–2493.

LIVINGSTONE JI, FILIPE MI AND WASTELL C. (1992). The poor prognosis of proximal compared to distal gastric cancer is not a function of tumour proliferation. *Br. J. Surg.*, **79**, 1255.

LIVINGSTONE JI, FILIPE MI AND WASTELL C. (1994). The expression of transforming growth factor alpha during experimental gastric carcinogenesis. *Gut*, **35**, 604–607.

MEYERS WC, DAMIANO RJ, POSTLETHWAIT RW AND ROTOLO FS. (1987). Adenocarcinoma of the stomach. Changing patterns over the last 4 decades. *Ann. Surg.*, **205**, 1–8.

MIWA K. (1979). Cancer of the stomach in Japan. *Gann Monographs Cancer Res.*, **22**, 61–75.

MIYACHI K, FRITZLER J AND TAN EM. (1978). Autoantibody to a nuclear antigen in proliferating cells. *J. Immunol.*, **121**, 2228–2234.

NAKAYAMA H, YASUI W, YOKOZAKI H AND TAHARA E. (1993). Reduced expression of nm23 is associated with metastasis of human gastric carcinomas. *Jpn. J. Cancer Res.*, **84**, 184–190.

PRELICH G AND STILLMAN B. (1988). Coordinated leading and lagging strand synthesis during SV40 DNA replication *in vitro* requires PCNA. *Cell*, **53**, 117–126.

ROWLANDS DC, BROWN HE, BARBER PC AND JONES EL. (1991). The effect of tissue fixation on immunostaining for proliferating cell nuclear antigen with the monoclonal antibody PC10. *J. Pathol.*, **165**, 356–357.

SASAKI K, TOMITA Y, AZUMA M, SHIDA S AND SIMIZU B. (1992). Amplification and over-expression of the c-*erb*B-2 proto- oncogene in human gastric cancer. *Gastroenterol. Jpn.*, **27**, 172–178.

SPORN MB AND TODARO GJ. (1980). Autocrine secretion and malignant transformation of cells. *N. Engl. J. Med.*, **302**, 878–880.

STEEG PS, BEVILACQUA G, KOPPER L, THORGEIRSSON UP, TALMADGE JE, LIOTTA LA AND SOBEL ME. (1988). Evidence for a novel gene associated with low tumour metastatic potential. *J. Natl Cancer Inst.*, **80**, 200–204.

SUGIYAMA K, YONEMURA Y AND MIYAZAKI I. (1989). Immunohistochemical study of epidermal growth factor and epidermal growth factor receptor in gastric carcinoma. *Cancer*, **63**, 1557–1561.

TAHARA E, SUMIYOSHI H, HATA J, YASUI W, TANIYAMA K, HAYASHI T, NAGAE S AND SAKAMOTO S. (1986). Human epidermal growth factor in gastric carcinoma as a biologic marker of high malignancy. *Jpn. J. Cancer Res. (Gann)*, **77**, 145–152.

TAHARA E, YOKOZAKI H AND YASUI W. (1993). Growth factors in gastric cancer. In: *Gastric cancer*, Nishi M, Ichikawa H, Nakajima T, Maruyama K and Tahara E (Eds). pp. 209–217. Springer-Verlag: Tokyo.

TAKAYOSHI N, MASATO I AND NAKAYAMA F. (1983). Changing state of gastric cancer in Japan. Histological perspective over the last 76 years. *Am. J. Surg.*, **145**, 226–233.

TAKEDA J, HASHIMOTO K, KOUFUJI K, KODAMA I, AOYAGI K AND KAKEGOWA T. (1992). A retrospective study of resected gastric cancers. *Kurume Med. J.*, **39**, 141–145.

TATEISHI M, TODA T, MINAMISONO Y AND NAGASAKI S. (1992). Clinopathological significance of c-*erb*B-2 protein expression in human gastric carcinoma. *J. Surg. Oncol.*, **49**, 209–212.

VAN DIERENDONCK JH, WIJSMAN JH, KEIJZER R, VAN DE VELDE CJH AND CORNELISSE CJ. (1991). Cell-cycle related staining patterns of anti- proliferating cell nuclear antigen monoclonal antibodies. Comparison with BrdUrd labelling and Ki-67 staining. *Am. J. Pathol.*, **138**, 1165–1172.

VINSON CR, SINGLER PB AND McKNIGHT SL. (1989). Scissors-grip model for DNA recognition by a family of leucine zipper proteins. *Science*, **246**, 911–916.

WOODS AL, HANBY AM, HALL PA, WASSEEM N, LANE DP AND LEVISON DA. (1990). The prognostic value of PCNA (proliferating cell nuclear antigen) immunostaining in gastrointestinal lymphomas. *J. Pathol.*, **161**, 342A.

YAMAMOTO T, IKAWA S, AKIYAMA T, SEMBA K, NOMURA N, MIYAJIMA N, SAITO T AND TOYOSHIMA K. (1986). Similarity of protein encoded by the human c-*erb*B2 gene to epidermal growth factor receptor. *Nature*, **319**, 231–234.

YASUI W, SUMIYOSHI H, HATA J, KAMEDA T, OCHIAI A, ITO H AND TAHARA E. (1988). Expression of epidermal growth factor receptor in human gastric and colonic carcinomas. *Cancer Res.*, **48**, 137–141.

YONEMURA Y, TAKAMURA H, NINOMIYA I, FUSHIDA S, TSUGAWA K, KAJI M, NAKAI Y, OHOYAMA S, YAMAGUCHI A AND MIYAZAKI I. (1992). Interrelationship between transforming growth factor-alpha and epidermal growth factor receptor in advanced gastric cancer. *Oncology*, **49**, 157–161.

YU C, HALL PA, FLETCHER CDM, CAMPLEJOHN R, WASSEEM N, LANE DP AND LEVISON DA. (1990). Immunohistochemical staining with a monoclonal antibody to proliferating cell nuclear antigen may be a good predictor of prognosis in haemangiopericytomas. *J. Pathol.*, **161**, 342A.

YU CC, FLETCHER CD, NEWMAN PL, GOODLAD JR, BURTON JC AND LEVISON DA. (1992). A comparison of proliferating cell nuclear antigen (PCNA) immunostaining, nucleolar organiser region (AgNOR) staining and histological grading in gastrointestinal stromal tumours. *J. Pathol.*, **66**, 147–152.